# Supplementary material for: Collaborative intelligence in AI: Evaluating the performance of a council of AIs on the USMLE
Source: PLOS Digit Health. 2025 Oct 9;4(10):e0000787. doi: 10.1371/journal.pdig.0000787 (PMC12510544; doi:10.1371/journal.pdig.0000787)
Supplement: S2 File — (ZIP) [file pdig.0000787.s002.zip › S2 Data Raw data files containing unprocessed question inputs and LLM outputs including transcripts of deliberations/STEP3_Question-15-1_Type-MC-NJ.docx]

**Facilitator** **[Round 1]:** A 32-year-old man comes to the office because of a 10-day history of a red rash that has spread over his entire torso. He says the rash is not itchy or painful. He reports having a flu-like illness nearly 5 weeks ago, with associated headache, neck stiffness, muffled hearing, and a canker sore on his tongue. All of these symptoms have resolved without treatment. Additional medical history is unremarkable and he takes no medications. BMI is 18 kg/m². Vital signs are normal. Specific additional history should be obtained to determine whether which of the following has occurred during the past 6 months? (A) Blood transfusions (B) Exposure to toxic chemicals (C) International travel (D) Tobacco use (E) Unprotected sexual intercourse

**AI_C:** RESTATING THE QUESTION: The inquiry here is to examine the necessary additional information to be collected from a 32-year-old patient. This man presented with a rash across his entire torso and had a recent history of a flu-like illness accompanied by a range of symptoms including headache, neck stiffness, muffled hearing, and a canker sore on the tongue. These symptoms resolved on their own without any medication. We are shown several options, and we must identify which one might be the cause of his recent health experiences during the past six months. These options include blood transfusions, exposure to toxic chemicals, international travel, tobacco use, or unprotected sexual intercourse. PLANNING: To start with, each of the propositions can potentially lead to health disorders but with varying possibilities based on the known symptoms. Blood transfusions can sometimes result in reactions and diseases but are less likely due to the absence of signs like fever, jaundice, or bleeding. Exposure to toxic chemicals could lead to a variety of health issues, rash included, but the chemical-specific pattern of symptoms is absent. Tobacco use mainly affects the respiratory system and is not strongly associated with his symptoms. Hence, the two most plausible assumptions would be: international travel leading to a potential exotic disease, or unprotected sexual intercourse resulting in a sexually transmitted disease. CLINICAL REASONING: The rash combined with flu-like symptoms, headache, neck stiffness, muffled hearing, and a mouth ulcer, seem to align more with a systemic infectious disease which could be obtained through international travel. The symptoms are not specific to any sexually transmitted diseases, which commonly manifest with genital ulcers, discharge, or urinary symptoms. The rash, being neither itchy nor painful, fits with a variety of systemic infectious diseases and also with secondary syphilis. Tertiary rash in syphilis can be asymptomatic. In this assumption, syphilis is a sexually transmitted infectious disease, which can also present with a rash and flu-like symptoms, thus aligning with the patient's clinical history. CONCLUDING: After analyzing the nature of symptoms, there is a relatively higher possibility of the patient having contracted an infectious disease as a result of unprotected sexual intercourse or international travel. However, considering the presence of an asymptomatic rash, a canker sore on the tongue, and non-specific systemic symptoms that resolved on their own, it seems more likely that the patient could be suffering from a sexually transmitted disease, such as Syphilis. RESPONSE: Therefore, to determine the likely cause of his medical condition during the past six months, the patient's history of unprotected sexual intercourse should be explored.

**AI_E:** RESTATING THE QUESTION: The question is asking us which aspect of the patient's history would be most helpful to ask about to get a better understanding of his current condition. The man is 32, has a rapidly spreading red rash over his torso which isn't associated with itchiness or pain, and has had a recent flu-like illness that resolved on its own. His medical history doesn't report anything of note, he takes no medications, his BMI is within the healthy range, and his vitals are normal. We're being asked to consider five possible areas to delve into: blood transfusions, exposure to toxic chemicals, international travel, tobacco use or unprotected sexual intercourse. Each of these could provide a clue to possible risk factors or exposures that may have contributed to his health issue. PLANNING: Assuming each of these events has the potential to cause his symptomatology, we need to allude to a reasonable explanation of how each could be involved in his clinical condition. The clinical significance of a history of blood transfusions could be related to possible transmission of infections, toxic exposures may have dermatological manifestations, international travel raises possibilities of exposure to infectious diseases, tobacco use can have a range of systemic effects, and unprotected sexual intercourse could indicate potential for sexually transmitted infections (STIs). Given his flu-like illness, comprehensive rash, and spontaneous resolution of symptoms, an infectious disease appears to be a strong candidate; moreover, an STI such as secondary syphilis needs to be considered. CLINICAL REASONING: Although each of these events could potentially contribute to his condition, the reported symptoms possess strong resemblance to those of secondary syphilis: a rash (often on the entire body), flu-like symptoms, a canker sore (possibly a syphilitic sore), and resolution of symptoms without treatment (as can occur in the early stages of syphilis). Secondary syphilis can present weeks to months after the initial infection, during which time the patient might not have any symptoms. The rash it causes is typically not itchy nor painful. Therefore, a recent history of unprotected sexual intercourse should be investigated to evaluate the risk of STIs. CONCLUDING: From the afore-described reasoning, it seems most plausible, considering his symptomatology and the common manifestations of secondary syphilis, to enquire about unprotected sexual intercourse. RESPONSE: Hence, to understand the most plausible etiology of this patient's condition and his rash, it would be most worthwhile to specifically inquire about any recent unprotected sexual intercourse (Option E), as this could indicate a risk for sexually transmitted infections such as syphilis.

**AI_B:** RESTATING THE QUESTION: This 32-year-old man presented with an all-over body rash for the past 10 days that's non-itchy and non-painful. About a month ago, he experienced flu-like symptoms, accompanied by headache, neck stiffness, muffled hearing, and a canker sore on his tongue, which all resolved without treatment. His past medical history is unremarkable, he takes no medication, and his BMI points to a normal weight for his height. His vital signs are also normal. We are required to discern which additional piece of information from his recent past (around 6 months) could help understand the cause of his symptoms. The options include: (A) any recent blood transfusions, (B) exposure to toxic chemicals, (C) any international travel, (D) tobacco use, or (E) unprotected sexual intercourse. PLANNING: Each option represents different assumptions that may be related to his symptoms. For instance, blood transfusions could point to an infection transmitted through the transferred blood, exposure to toxic chemicals might suggest a skin reaction or systemic toxicity, international travel could hint at a disease acquired from another region, tobacco use could potentially relate to a variety of systemic health issues, and unprotected sexual intercourse might indicate a sexually transmitted infection (STI). CLINICAL REASONING: Given his symptoms and their timeline, it's important to prioritize which assumption is most likely. His rash, combined with his history of flu-like symptoms, headache, neck stiffness, muffled hearing, and the canker sore all seem to point towards some sort of infectious process. The rash specifically, and its progression, coupled with his prior symptoms, suggest a systemic infection that could potentially be sexually transmitted rather than symptoms related to chemical exposure or tobacco use. Blood transfusions could cause similar symptoms if he had received an infected blood, but no such history is mentioned. Likewise, while certain illnesses can be picked up traveling, there's nothing specifically indicating that the patient has travelled recently. CONCLUDING: Considering the timeline and description of the patient's symptoms, including the rash, flu-like symptoms, and oral sores, a sexually transmitted disease, such as secondary syphilis, could be a viable concern. Secondary syphilis often presents with a non-itchy rash that can involve the entire body, and may occur several weeks after initial flu-like symptoms. So, his sexual history could potentially reveal a probable cause. RESPONSE: It seems essential to consider option (E) Unprotected sexual intercourse as the appropriate domain to delve further into. The pattern and progression of the patient's symptoms suggest it might be attributable to a sexually transmitted disease, namely syphilis, which can elicit the symptoms he presents.

**AI_A:** 1) RESTATING THE QUESTION: The question is about a 32-year-old male presenting with a spreading red rash on his torso. Prior to this, he reports experiencing a flu-like illness with a few specific symptoms. His medical history is generally well, with no medications in use. His BMI is 18 kg/m² and vitals are within normal ranges. The aim here is to determine what additional part of his history we need to explore to understand better what may have happened to him over the past six months. The options range from blood transfusions, exposure to toxic chemicals, international travel, tobacco use, or unprotected sexual intercourse. 2) PLANNING: Given the symptoms and their progression, there are multiple potential causes to consider. For instance, blood transfusions may carry the risk of transmissible diseases, which could lead to skin manifestations. Exposure to toxic chemicals might be linked to dermatological complications. International travel may expose the patient to different pathogens. Tobacco use could cause systemic issues leading to rashes. Finally, unprotected sexual intercourse raises the risk of sexually transmitted infections (STI), which could have cutaneous (skin) manifestations. 3) CLINICAL REASONING: This requires the evaluation of several assumptions about the potential diagnoses. The presentation of a red rash, flu-like illness, and the noticeable but resolved symptoms such as headache, neck stiffness, muffled hearing, and a canker sore could indicate a systemic infection, possibly of a viral nature. Importantly, many of these symptoms are non-specific, indicating a need for more targeted investigation. Several viruses such as the Herpes group, HIV, or Hepatitis B and C, which are acquired through blood transfusions or unprotected sexual intercourse can present with a similar clinical picture. It seems less likely that the cause is related to toxic chemicals, tobacco, or international travel based on the presented symptoms. 4) CONCLUDING: While all five possibilities warrant a detailed patient history, the most prudent for the current clinical scenario seems to be uncovered details about past blood transfusions or unprotected sexual intercourse. Further questioning could reveal whether the person had a possibility of exposure to viral infections such as HIV, Herpes group, Hepatitis B, or C. 5) RESPONSE: Based on the patient's history, specific additional history should be obtained to determine whether unprotected sexual intercourse (E) took place during the past six months. This route of transmission is a crucial factor to consider given the clinical manifestation of non-itchy, painless red rash along with a history suggestive of a systemic illness. Secondary syphilis (caused by Treponema pallidum), for example, can present in a similar way. If positive, the person might require further diagnosis and treatment for sexually transmitted infections (STIs).

**AI_D:** (1) RESTATING THE QUESTION: The issue under review involves a 32-year-old man who presents with a non-itchy, non-painful red rash that has been present for 10 days and covers his entire torso. The patient had a set of symptoms associated with a flu-like illness — including a headache, neck stiffness, muffled hearing, and a canker sore on his tongue — approximately 5 weeks prior. These symptoms, however, have all resolved without any intervention. The patient's additional medical history is unremarkable; he does not take any medications, and his body mass index (BMI) is 18 kg/m², which is within the healthy range. His vital signs are also normal. The question is, which further information from his history is necessary to determine whether any of the following events have taken place within the last six months: (A) Blood transfusions (B) Exposure to toxic chemicals (C) International travel (D) Tobacco use (E) Unprotected sexual intercourse (2) PLANNING: Given the patient's past flu-like illness and the existence of a rash, international travel (C) and unprotected sexual intercourse (E) are two assumptions that could provide potential etiological basis for his condition. Blood transfusion (A) and exposure to toxic chemicals (B) may be less likely given his current clinical picture. Tobacco use (D) does not fit with this patient’s particular presentation. Among the top two assumption scenarios, the unprotected sexual intercourse history should be clinically prioritized for further exploration because it could likely explain both the flu-like prodromal symptoms and rash, depending on the type of sexually transmitted infection (STI). (3) CLINICAL REASONING: Conducting additional inquiry in relation to unprotected sexual intercourse is clinically relevant given the potential for sexually transmitted infections (STIs). Flu-like symptoms, a common early manifestation of certain STIs (such as primary syphilis or HIV), were reported by the man around five weeks ago alongside a singular canker sore on the tongue; the latter is a common site of chancres or herpes. These symptoms have all spontaneously resolved, which is typical with the natural course of such infections. The non-itchy, widespread rash could be associated with the secondary stage of syphilis or acute HIV, which often occurs weeks after the initial signs of infection. (4) CONCLUDING: Thus, obtaining a comprehensive sexual history from a patient who has presented with a widely spread rash alongside a history of a flu-like illness could yield potentially valuable information. Given the overlap in symptoms, this patient could have been exposed to a sexually transmitted infection such as syphilis or HIV. Both illnesses can manifest early flu-like symptoms that later transition into an asymptomatic period, followed by further clinical manifestations such as a rash. (5) RESPONSE: Therefore, further investigation into whether the patient has had unprotected sexual intercourse (E) within the past six months should be considered. The aim is to identify potential infection risks and commence subsequent interventions based on the findings.

**Facilitator** The consensus among the AI responses is that the most relevant additional history to explore for the 32-year-old male patient presenting with a spreading red rash on his torso and a recent flu-like illness is unprotected sexual intercourse (Option E). This conclusion is based on the patient's symptoms, which include a non-itchy, non-painful rash, flu-like symptoms, and a canker sore on his tongue, all of which have resolved without treatment. These symptoms suggest a systemic infection, possibly of a viral nature, and align with the clinical picture of a sexually transmitted infection (STI), such as secondary syphilis. While other options like blood transfusions, exposure to toxic chemicals, international travel, and tobacco use could potentially contribute to his condition, they seem less likely based on the presented symptoms. Therefore, further investigation into the patient's sexual history is recommended to identify potential infection risks and commence subsequent interventions based on the findings.
